# Two Types of Receptive Field Dynamics in Area V4 at the Time of Eye Movements?

**DOI:** 10.3389/fnsys.2017.00013

**Published:** 2017-03-21

**Authors:** Till S. Hartmann, Marc Zirnsak, Michael Marquis, Fred H. Hamker, Tirin Moore

**Affiliations:** ^1^Department of Neurobiology, Harvard Medical SchoolBoston, MA, USA; ^2^Department of Neurobiology and Howard Hughes Medical Institute, Stanford University School of MedicineStanford, CA, USA; ^3^Department of Computer Science, Chemnitz University of TechnologyChemnitz, Germany

**Keywords:** attention, eye movements, extrastriate cortex, receptive field, remapping

## Abstract

How we perceive the world as stable despite the frequent disruptions of the retinal image caused by eye movements is one of the fundamental questions in sensory neuroscience. Seemingly convergent evidence points towards a mechanism which dynamically updates representations of visual space in anticipation of a movement (Wurtz, 2008). In particular, receptive fields (RFs) of neurons, predominantly within oculomotor and attention related brain structures (Duhamel et al., 1992; Walker et al., 1995; Umeno and Goldberg, 1997), are thought to “remap” to their future, post-movement location prior to an impending eye movement. New studies (Neupane et al., 2016a,b) report observations on RF dynamics at the time of eye movements of neurons in area V4. These dynamics are interpreted as being largely dominated by a remapping of RFs. Critically, these observations appear at odds with a previous study reporting a different type of RF dynamics within the same brain structure (Tolias et al., 2001), consisting of a shrinkage and shift of RFs towards the movement target. Importantly, RFs have been measured with different techniques in those studies. Here, we measured V4 RFs comparable to Neupane et al. (2016a,b) and observe a shrinkage and shift of RFs towards the movement target when analyzing the immediate stimulus response (Zirnsak et al., 2014). When analyzing the late stimulus response (Neupane et al., 2016a,b), we observe RF shifts resembling remapping. We discuss possible causes for these shifts and point out important issues which future studies on RF dynamics need to address.

## Introduction

Investigating visual representations around the time of saccadic eye movements, Neupane et al. (2016a,b) argue that receptive fields (RFs) of neurons within area V4 predominately exhibit a certain type of dynamics, consisting of a shift of RFs to their post-movement location (Duhamel et al., 1992; Walker et al., 1995; Umeno and Goldberg, 1997), referred to as “future field (FF) remapping”. These observations appear to be at odds with an earlier study, in which Tolias et al. (2001) reported a different type of V4 RF dynamics, consisting of a shrinkage and shift of RFs towards the saccade target (ST), referred to as “saccade target shifts”. Importantly, whereas the observed RF dynamics reported by Neupane et al. (2016a,b) occur long after the offset of the eye movement, the observed RF dynamics reported by Tolias et al. (2001) occur just around the onset of the movement.

Another difference between the studies is the way RFs were measured. Whereas Tolias et al. (2001) measured the RFs with visual probes continuously present throughout the period of the eye movement, Neupane et al. (2016a,b) measured RFs by briefly flashing visual probes shortly before the movement. Furthermore, the latter analysis of RF dynamics seems to neglect the immediate neuronal responses evoked by the probe, and instead focuses on responses with unusually high latencies (>100 ms; Schmolesky et al., 1998) occurring well after the completion of the eye movement.

We measured V4 RFs with a protocol comparable to Neupane et al. (2016a,b). For the immediate, early neuronal responses to presaccadic visual probes we observed a shrinkage and shift of RFs towards the ST consistent with Tolias et al. (2001). For the later, post-movement part of the responses we observed RF changes resembling FF shifts consistent with Neupane et al. (2016a,b). We discuss possible causes for these observed RF shifts.

## Materials and Methods

All animal procedures complied with the National Institutes of Health Guide for Care and Use of Laboratory Animals and were approved by the Harvard Medical Area Standing Committee on Animals.

We recorded responses of neurons within extrastriate cortex (V4) of the macaque monkey (*Macaca mulatta*) from a permanently implanted 96-channel multielectrode array (Utah array, Blackrock Microsystems, Salt Lake City, UT, USA). For each channel we set a voltage threshold and stored the times when the voltage crossed the threshold. We analyzed multi-unit activity from channels that were responsive to the probe stimulus during fixation (*n* = 71/96 units).

**Figure 1:**
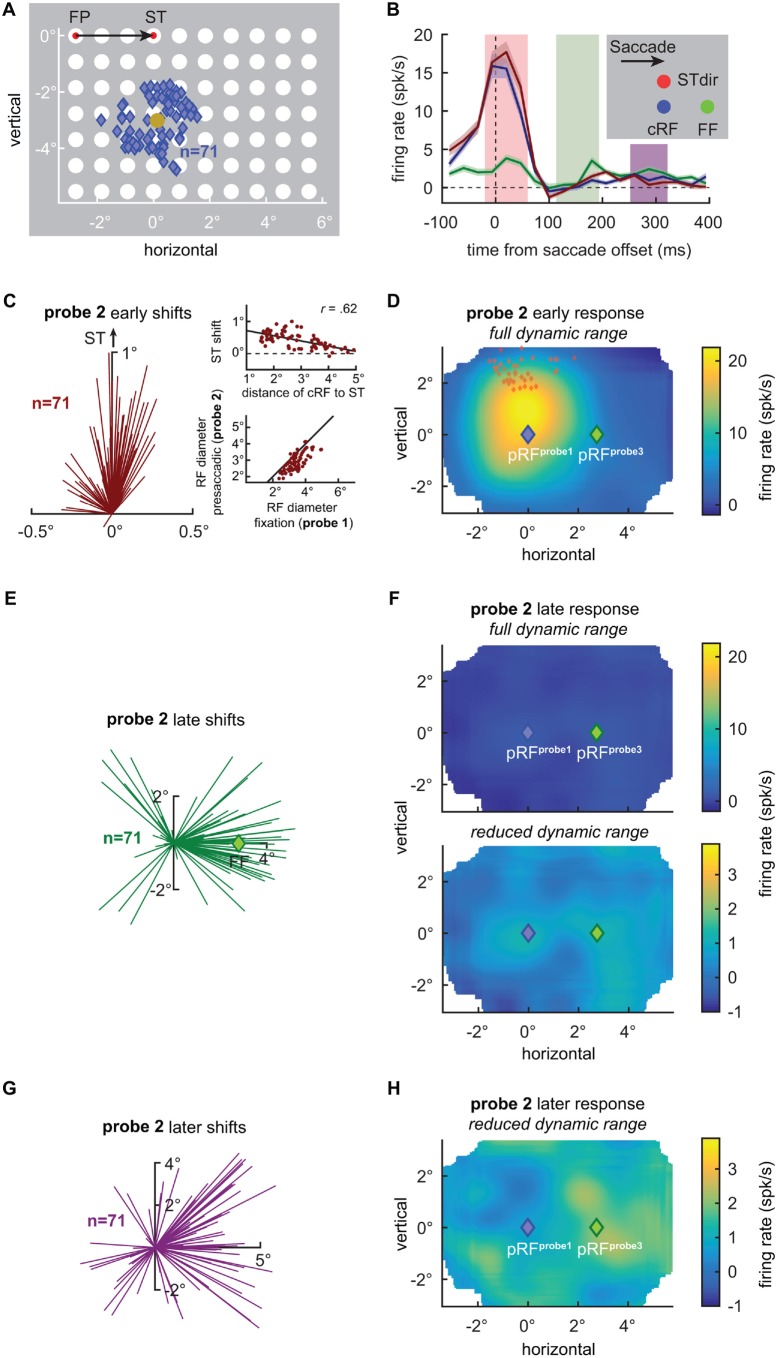
**V4 receptive field shifts at the time of saccades. (A)** Centers of 71 V4 current receptive fields (cRFs) plotted together with the visual probe grid (white disks) drawn to scale. Responses of V4 neurons were probed during fixation at the fixation point (FP) or the saccade target (ST; red disks), long before and after an eye movement, or shortly before an eye movement from the FP to the ST. RFs were then estimated for each condition by fitting Gaussian functions at various times to the neuronal responses recorded in the three conditions. Blue diamonds indicate the centers of the estimated cRFs during fixation at the FP long before and after an eye movement (see “Materials and Methods” Section for details). The average cRF center was *x* = 0.15° and *y* = −3.07° (gold disk) relative to the ST (*x* = 0°, *y* = 0°). **(B)** Mean responses of the recorded V4 population to three probes flashed briefly before the onset of a saccade (probe 2). The blue line shows the mean response to a probe (blue disk) presented closest to the centers of the individual cRFs. The red line shows the mean response to a probe (red disk) presented 0.9° above the probe closest to the cRF, closer to the ST (STdir). The green line shows the mean response to a probe (green disk) presented closest to the centers of the estimated future fields (FFs). **(C)** RF shift estimates based on the immediate, early presaccadic probe responses (red shaded area in **B**). Each line indicates the difference between the center of the cRF (*x* = 0°, *y* = 0°), as measured long before an eye movement, and the center of the RF, as measured shortly before movement onset (alignment as in Tolias et al., 2001). Consistent with Tolias et al. (2001), V4 RF centers shifted towards the ST (see also Figure 2I). The amplitude of the RF shift towards the ST depended on the distance of the cRF center to the ST (upper inset). Solid line depicts best linear fit. Furthermore, consistent with Tolias et al. (2001) V4 RFs shrank by relative to their cRF size (lower inset). Solid line depicts line of unity. **(D)** Average population RF (pRF) based on the early visually evoked activity relative to saccade offset, as measured shortly before a saccade (probe 2) during fixation at the FP (see “Materials and Methods” Section for details). Full dynamic range of responses is shown. Consistent with individual RFs **(C)**, the average RF shifts upwards, away from the current population RF (pRF) center as measured during fixation (probe 1; blue diamond; Figure 2A) and towards the STs (red diamonds; see also Figure 2I). **(E)** RF shift estimates based on the late, post-movement activity of presaccadic probe responses (green shaded area in **B**). RF centers shift in the direction of the FF (green diamond at *x* = 2.76°) consistent with Neupane et al. (2016a,b); see also Figure 2J. **(F)** Average pRF based on the late activity relative to saccade offset. The full dynamic range of responses is shown in the top panel. The bottom panel shows a strongly reduced dynamic range. Consistent with individual RFs **(E)**, the average RF shifts rightwards, away from the pRF center as measured during fixation (probe 1; blue diamond) and towards the FF. **(G)** RF shift estimates based on the later, post-movement activity of presaccadic probe responses (purple shaded area in **B**). Each line indicates the difference between the center of the cRF (*x* = 0°, *y* = 0°), as probed (probe 1) long before an eye movement during fixation at the FP, and the center of the RF as probed (probe 2) shortly before movement onset. RF centers shift into the direction of the FF (*x* = 2.76°, *y* = 0°; see also Figure 2K). **(H)** Average pRF based on the later activity relative to saccade offset. Reduced dynamic range of responses is shown. The activity is still biased to the right, resembling the activity pattern shown in Figure 2B.

The flashed probe stimuli, white disks (0.5° diameter, 15 cd/m^2^), were presented against a gray background (1.2 cd/m^2^; Figure 1A) on a CRT screen with a 75 Hz refresh rate, positioned 75 cm in front of the animal in a normally illuminated room. The animal received a reward for successfully fixating at the fixation point (FP; red disk, 0.1° diameter), then performing a saccade to the ST (red disk, 0.1° diameter; step task), which was positioned 2.75° to the right and 0.25° above the FP resulting in a nominal saccade amplitude of 2.76° (average empirical saccade amplitude was 2.65°), comparable to Neupane et al. (2016b). During a trial, we presented three probes (see below). The ST location was chosen to dissociate FF shifts and ST shifts by 90° (Hamker et al., 2008; Zirnsak et al., 2010, 2014). The probe was presented for one monitor frame (13.3 ms) at one of 70 locations tiling the space surrounding the recorded units’ RFs. The probes were arranged in a 7 × 10 rectangular grid with the longer dimension parallel to the saccade vector. Note, for the sake of clarity, all figures, display methods, and results after rotated by 5.2° as if the saccades were made purely horizontally.

We analyzed the responses to the probes long before (<−500 ms, probe 1), immediately before (−100 ms to 0 ms, probe 2), and long after (>500 ms, probe 3) a saccadic eye movement, as in Neupane et al. (2016a,b). The first probe in that sequence was randomly flashed between 200 ms and 400 ms after the initial fixation, the movement cue (step) was given randomly 900–1400 ms after initial fixation, and the last probe 3 was flashed 1700–2400 ms after initial fixation. Saccade onset and offset were detected using Friedman and Priebe (1998) latency estimation adjusted for eye movements, visually inspected for accuracy, and corrected by the measured latency (14 ms) of the optical eye tracker (ISCAN, Woburn, MA, USA). We estimated response maps for each unit by summing the spikes recorded for various time bins. To estimate RF location and size, we fit two-dimensional Gaussian functions to the response map. Each Gaussian function had six parameters: *x* and *y* location, *x* and *y* size (sigma), peak firing rate and baseline firing rate. These fits provide an RF center estimate unbiased by changes in baseline activity. Note, all reported results hold true when using a center of mass method to estimate RF centers as in Neupane et al. (2016a,b). We also computed an average population activity map (pRF). For each individual unit, we linearly interpolated a given response map and aligned it to the cRF center. We averaged the aligned maps of all units, excluding map locations where less than half the units contributed firing rate estimates.

## Results

**Figure 2:**
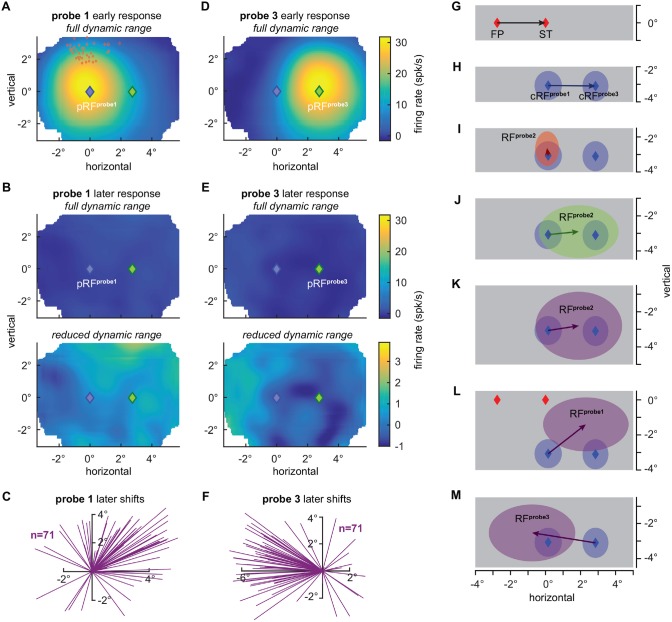
**V4 receptive field shifts during fixation and at the time of saccades. (A)** Average population RF (pRF) based on early visually evoked activity (53–106 ms) relative to probe onset (probe 1), as measured long before a saccade during fixation at the FP (see “Material and Methods” Section for details). Blue diamond indicates the center of all RFs (pRF^probe1^). Red diamonds indicate the STs relative to the individual current RF (cRF) centers (Figure 1A). Full dynamic range of responses is shown. **(B)** Average RF based on later visually evoked activity relative to probe onset (249–347 ms) during fixation of the FP. The full dynamic range of responses is shown in the top panel. The bottom panel shows a strongly reduced dynamic range. The activity is shifted to the right resembling a “negative RF”. **(C)** RF shift estimates based on the later responses to fixation probes. Each line indicates the difference between the center estimated with the Gaussian fit of the cRF (*x* = 0°, *y* = 0°), as measured long before an eye movement during fixation at the FP and based on the immediate probe response, and the center of the RF based on the later probe responses. RF centers shift to the right into the direction of the FF (*x* = 2.76°, *y* = 0°) and upward with an average amplitude of 2.72° (*p* < 10^−7^, Wilcoxon rank test; see also **L**). **(D)** Average RF based on early visually evoked activity (53–106 ms) relative to probe onset (probe 3), as measured long after a saccade during fixation at the ST. Green diamond indicates the center of all RFs (pRF^probe3^). Full dynamic range of responses is shown. **(E)** Population RF based on the later visually evoked activity (249–347 ms) relative to probe onset during fixation at the ST. The full dynamic range of responses is shown in the top panel. The bottom panel shows a strongly reduced dynamic range. This time the activity is shifted to the left resembling again a “negative RF”. **(F)** RF shift estimates based on the later responses to fixation probes. Each line indicates the difference between the center of the cRF (*x* = 0°, *y* = 0°), as measured long after an eye movement during fixation and based on the early probe response, and the center of the RF based on the later probe responses. RF centers shift to the left into the direction of the pre eye movement cRF as measured during fixation at the FP with an average amplitude of 3.66° (*p* < 10^−10^, Wilcoxon rank test; see also **M**). **(G)** Nominal saccade vector (2.76°) from the FP to the ST (*x* = 0°, *y* = 0°). **(H)** Average displacement (2.70°) of current RF (cRF) centers as measured long before and after a saccade during fixation at the FP (probe 1; *x* = 0.15°, *y* = −3.07°) and at the ST (probe 3; *x* = 2.85°, *y* = −3.1°). cRF estimates are based on the immediate, early probe responses **(A,D)**. Shaded regions depict one standard deviation around the respective means. **(I)** Average shift of RF centers (0.41°) towards (*x* = 0.09°, *y* = −2.67°) the ST based on the immediate, early responses to presaccadic probes (probe 2; Figure 1B). Shaded regions depict one standard deviation around the respective means. **(J)** Average shift of RF centers (1.75°) into the FF (cRF^probe3^) direction to the right (*x* = 1.9°, *y* = −2.9°). RF estimates are based on the late presaccadic probe (probe 2) responses (Figure 1B). Shaded regions depict one standard deviation around the respective means. **(K)** Average shift of RF centers (1.77°) into the direction of the FF (*x* = 1.9°, *y* = −2.7°). RF estimates are based on the later responses to presaccadic probes (Figure 1B). Shaded regions depict one standard deviation around the respective means. **(L)** Average shift of RF centers (2.72°) into the FF direction and upwards away from the ST (*x* = 2.3°, *y* = −1.4°). RF estimates are based on the later probe (probe 1) responses during fixation at the FP **(B)**. Shaded regions depict one standard deviation around the respective means. **(M)** Average shift of RF centers (3.66°) into the direction of the cRF^probe1^ (*x* = −0.77°, *y* = −2.53°). RF estimates are based on the later probe (probe 3) responses during fixation at the ST **(E)**. Shaded regions depict one standard deviation around the respective means.

First, we compared the RFs as measured long before a saccade (probe 1) to the RFs as measured long after a saccade (probe 3). To do so, we choose a time window of 53–106 ms, including the majority of the visually evoked response, after probe onset, to estimate the RFs of the two fixation conditions. As expected for V4 neurons, RFs followed a retinocentric organization between fixations, with an average distance (2.70°) between the RF centers close to the true displacement (2.76°) of the FP (Figures 2A,D,G,H). In contrast, Neupane et al. (2016a) estimates for the RF displacement between the two fixation conditions seem to be considerably less than the actual displacement of the FP (20°), with an average of only about 13°–14° judging by their figures two and three. This underestimation might be indicative of a systematic tendency of the RF center estimate in Neupane et al. (2016a) towards the center of the probe grid (below).

Second, to investigate RF shifts at the time of the eye movement, we calculated the response maps for the time window from −20 ms to 60 ms aligned to the saccade offset which included most of the stimulus evoked responses. Note, given the stimulation protocol as described above and the range of visual latencies of V4 neurons (e.g., Schmolesky et al., 1998) the majority of the neuronal activity evoked by a presaccadic probe is expected to occur after the movement onset. We found that the immediate visual responses evoked by the probe presented before the movement (probe 2) were stronger for probes presented closer to the ST when compared with the response to a probe presented at the cRF center as measured long before the saccade (probe 1; Figure 1B, red line above blue line, *p* < 0.02, Wilcoxon rank test) as predicted by models of perisaccadic visual processing (Hamker and Zirnsak, 2006; Hamker et al., 2008) and consistent with the notion that attention is locked at the ST before movement onset (reviewed in Zhao et al., 2012; Moore and Zirnsak, 2017). Furthermore, RF centers estimated for the visually evoked response right around the time of the saccade shifted towards the ST (Figure 1C; *p* < 10^−10^, Rayleigh test; Figures 1D, 2H) consistent with Tolias et al. (2001). The size of this shift depended on the cRF center distance to the ST (Figure 1C, upper inset). The average RF shift amplitude of 0.41° (*p* < 10^−10^, Wilcoxon rank test) amounted to 38% of the average RF shift reported by Tolias et al. (2001) and to 150% of the average RF shift reported by Connor et al. (1997), who measured V4 RF shifts during covert attention, when accounting for the cRF diameter. In addition to the RF shift, and consistent with observations by Tolias et al. (2001), we also observed a significant shrinkage of RFs (14.5%, *p* < 10^−10^, Wilcoxon signed rank test; Figure 1C, lower inset). This average shrinkage amounts to 80% of the average RF shrinkage reported by Tolias et al. (2001). We wondered whether a potential bias of RF center estimates towards the center of the probe grid (see above) might have obscured shifts towards the ST for RFs based on the early visual evoked responses in Neupane et al. (2016a,b). Moreover, the extremely high contrast of the probes used in the majority of their measurements might have prevented Neupane et al. (2016a,b) from observing changes of RFs based on the immediate probe responses as well. That is, perisaccadic RF changes have been linked to gain modulation mechanisms (Hamker and Zirnsak, 2006) which might be ineffective for extremely high contrast probes (Hamker et al., 2008). Neupane et al. (2016a,b) used white probes with a luminance of 22.5 cd/m^2^ relative to a dark background with a luminance of <0.01 cd/m^2^. These luminance values result in a peak stimulus contrast of >2249 (Weber) and of >99.9% (Michelson). Neupane et al. (2016a,b) report a reduction of the probe luminance, delivered by a CRT video projector, of 99% 6 ms after the nominal probe offset. This means a residual probe luminance of 0.225 cd/m^2^, roughly 300–1000 times the reported luminance detection threshold of dark adapted observers (Georg et al., 2008), which equals a probe contrast of >21.5 (Weber) and >91.5% (Michelson). These residual probe contrasts in Neupane et al. (2016a,b) are higher than the peak values used in our measurements (11.5 Weber, 85.2% Michelson).

Finally, we tested for RF shifts long after the saccade, as reported by Neupane et al. (2016a,b). At roughly 100–200 ms after saccade offset we observed higher responses to probes presented inside the FF, as compared to the cRF and the STdir probe location (*p* < 0.005, Wilcoxon rank test; Figure 1B). Furthermore, although much smaller in size as compared to the earlier responses (Figures 1B,F) and based on significantly poorer fits (*p* < 10^−10^, Wilcoxon rank test; mean *R*^2^ = 0.13, min *R*^2^ = 0.07, max *R*^2^ = 0.70 compared to mean *R*^2^ = 0.86, min *R*^2^ = 0.68, max *R*^2^ = 0.94 of the RF fits based on the early responses), estimates of RF centers based on the late response lead to a systematic bias (*p* < 10^−4^, Rayleigh test) towards the FF (Figures 1E, 2J), with an average size of 1.75° (*p* < 10^−6^, Wilcoxon signed rank test). These observations are consistent with Neupane et al. (2016a,b). However, although the contrast of our stimuli was considerably lower than the contrast of the stimuli used by Neupane et al. (2016a,b) to map RFs, we cannot rule out an influence of a stimulation artifact on these late responses and RF shifts. That is, these late shifts are based on responses long after the completion of the eye movement at a time where V4 neurons have been reported to be responsive again to stimuli presented within their postsaccadic RF, that is the presaccdic FF (Tolias et al., 2001). This means that a decaying phosphor trace (stimulation was done by means of a CRT as in Neupane et al., 2016a,b; see above) of presaccadic probes presented at the FF location could have fallen inside the RF after the eye movement and stimulated the neurons postsaccadically (see also Jonides et al., 1982, 1983). This possibility would lead to observations resembling late FF shifts. For even later parts of the response with respect to saccade offset, Neupane et al. (2016a) reported an increasing tendency of RF shifts towards the ST. We failed to find this later ST shift in our data. Instead the activity for responses later than 250 ms after saccade offset still exhibited a bias towards the right part of the probe grid and RF centers are on average mainly shifted into the saccade direction with an amplitude of 1.77° (*p* < 10^−6^, Wilcoxon signed rank test; Figures 1G,H, 2K). Interestingly, however, we also observe a FF shift (probe 1) during stable fixation (FP) in the absence of any eye movements. This FF shift is caused by a reversal of the late responses resulting in a “negative RF” (Figures 2B,C,L) and is reversed, based on the geometry of our stimulus grid, for RFs measured during stable fixation at the ST (probe 3; Figures 2E,F,M).

## Discussion

In summary, when analyzing RFs based on the immediate response to probes presented briefly before a saccade, we observed a shrinkage and shift of RFs towards the ST, consistent with the observations reported by Tolias et al. (2001), and consistent with models of perisaccadic visual processing (Hamker and Zirnsak, 2006; Hamker et al., 2008; Zirnsak et al., 2010). When analyzing RFs based on late responses well after saccade offset as in Neupane et al. (2016a,b), we observed RF shifts in the direction of the FF. Importantly, these shifts occurred at a time when V4 neurons are observed to already respond to stimuli located in their postsaccadic RF when using continuous visual stimulation (Tolias et al., 2001). Thus, and as stated above, given our stimulation protocol we cannot exclude an influence of a decaying phosphor trace of the stimulus on these late responses, which could have stimulated the RF after the movement offset. Finally, we also observed a shift of RFs towards the FF in the absence of any eye movements. This observation was driven by a reversal of the later part of the visual responses of V4 neurons and the exact direction of the shift depended on the geometry of our probe grid.

In conclusion, two types of RF dynamics have been proposed to play an important part in maintaining a stable perception across eye movements: FF shifts and ST shifts. Future studies addressing the nature and function of those RF dynamics will be crucial in elucidating the neural basis of naturalistic vision in primates (Wurtz, 2008; Zirnsak and Moore, 2014). These studies also must eliminate alternative explanations of the observed RF dynamics.

## Author Contributions

TSH and MM designed and performed the experiment. TSH, MZ and MM designed and performed analyses. TSH, MZ, TM, FHH and MM wrote the manuscript.

## Funding

This work was supported by US National Institutes of Health grants T32 NS007484 and F32 EY025523 (TSH), T32 MH020017 (MM), R01 EY011379, the Core Grant for Vision Research P30 EY12196, BMBF 01GQ1409 (FHH), and R01 EY014924 (TM).

## Conflict of Interest Statement

The authors declare that the research was conducted in the absence of any commercial or financial relationships that could be construed as a potential conflict of interest.

## References

[B1] Connor E. C. Preddie D. C. Gallant J. L. Van Essen D. C. (1997). Spatial attention effects in macaque area V4. J. Neurosci. 17, 3201–3214. 9096154 10.1523/JNEUROSCI.17-09-03201.1997 PMC6573654

[B2] Duhamel J. R. Colby C. L. Goldberg M. E. (1992). The updating of the representation of visual space in parietal cortex. Science 255, 90–92. 10.1126/science.1553535 1553535

[B3] Friedman H. S. Priebe C. E. (1998). Estimating stimulus response latency. J. Neurosci Methods. 83, 185–194. 10.1016/s0165-0270(98)00075-2 9765132

[B4] Georg K. Hamker F. H. Lappe M. (2008). Influence of adaptation state and stimulus luminance on peri-saccadic localization. J. Vis. 8:15. 10.1167/8.1.15 18318618

[B5] Hamker F. H. Zirnsak M. (2006). V4 receptive field dynamics as predicted by a systems-level model of visual attention using feedback from the frontal eye field. Neural Netw. 19, 1371–1382. 10.1016/j.neunet.2006.08.006 17014990

[B6] Hamker F. H. Zirnsak M. Calow D. Lappe M. (2008). The peri-saccdic perception of objects and space. PLoS Comput. Biol. 4:e31. 10.1371/journal.pcbi.0040031 18282086 PMC2242822

[B8] Jonides J. Irwin D. E. Yantis S. (1982). Integrating information from successive fixations. Science 215, 192–194. 10.1126/science.7053571 7053571

[B7] Jonides J. Irwin D. E. Yantis S. (1983). Failure to integrate information from successive fixations. Science 222:188. 10.1126/science.6623072 6623072

[B9] Moore T. Zirnsak M. (2017). Neural mechanisms of selective visual attention. Ann. Rev. Psychol. 108, 47–72. 10.1146/annurev-psych-122414-033400 28051934

[B11] Neupane S. Guitton D. Pack C. C. (2016a). Two distinct types of remapping in primate cortical area V4. Nature Commun. 7:10402. 10.1038/ncomms10402 26832423 PMC4740356

[B10] Neupane S. Guitton D. Pack C. C. (2016b). Dissociation of forward and convergent remapping in primate visual cortex. Curr. Biol. 26, 491–492. 10.1016/j.cub.2016.04.050 27326707

[B12] Schmolesky M. T. Wang Y. Hanes D. P. Thompson K. G. Leutgeb S. Schall K. D.. (1998). Signal timing across the macaque visual system. J. Neurophysiol. 79, 3272–3278. 9636126 10.1152/jn.1998.79.6.3272

[B13] Tolias A. S. Moore T. Smirnakis S. M. Tehovnik E. J. Siapas A. G. Schiller P. H. (2001). Eye movements modulate visual receptive fields of V4 neurons. Neuron 29, 757–767. 10.1016/s0896-6273(01)00250-1 11301034

[B14] Umeno M. M. Goldberg M. E. (1997). Spatial processing in the monkey frontal eye field. I. Predictive visual responses. J. Neurophysiol. 78, 1373–1383. 9310428 10.1152/jn.1997.78.3.1373

[B15] Walker M. F. Fitzgibbon E. J. Goldberg M. E. (1995). Neurons in the monkey superior colliculus predict the visual result of impending saccadic eye movements. J. Neurophysiol. 73, 1988–2003. 7623096 10.1152/jn.1995.73.5.1988

[B16] Wurtz R. H. (2008). Neuronal mechanisms of visual stability. Vision Res. 48, 2070–2089. 10.1016/j.visres.2008.03.021 18513781 PMC2556215

[B17] Zhao M. Gersch T. M. Schnitzer B. S. Dosher B. A. Kowler K. (2012). Eye movements and attention: the role of pre-saccadic shifts of attention in perception, memory and the control of saccades. Vision Res. 74, 40–60. 10.1016/j.visres.2012.06.017 22809798 PMC3623695

[B19] Zirnsak M. Lappe M. Hamker F. H. (2010). The spatial distribution of receptive field changes in a model of peri-saccadic perception: predictive remapping and shifts towards the saccade target. Vision Res. 50, 1328–1337. 10.1016/j.visres.2010.02.002 20152853

[B18] Zirnsak M. Moore T. (2014). Saccades and shifting receptive fields: anticipating consequences or selecting targets. Trends Cogn Sci. 18, 621–628. 10.1016/j.tics.2014.10.002 25455690 PMC4279245

[B20] Zirnsak M. Steinmetz N. A. Noudoost B. Xu K. Z. Moore T. (2014). Visual space is compressed in prefrontal cortex before eye movements. Nature 507, 504–507. 10.1038/nature13149 24670771 PMC4064801

